# Dual Identity and Prejudice: The Moderating Role of Group Boundary Permeability

**DOI:** 10.3389/fpsyg.2017.00195

**Published:** 2017-02-16

**Authors:** Yuanyuan Shi, Jianning Dang, Wenwen Zheng, Li Liu

**Affiliations:** Beijing Key Lab of Applied Experimental Psychology, School of Psychology, Beijing Normal UniversityBeijing, China

**Keywords:** dual identity, group boundary permeability, intergroup prejudice, rural-to-urban migrants, urbanization policies

## Abstract

Past work suggested that dual identity was effective to reduce prejudice. This study extended research on dual identity and prejudice by identifying a boundary condition in this relationship, that is, group permeability. In Study 1, we replicated previous studies with Chinese individuals and found that inducing dual identity (emphasizing subgroup differences and a common nation identity), compared to the control condition, decreased the urban residents’ prejudice against rural-to-urban migrants. In Study 2, we manipulated the group boundary permeability using the *Hukou* system reform, and found that when the group boundary was permeable, dual identity was effective in reducing prejudice against rural-to-urban migrants. However, this effect vanished in the condition where the group boundary was impermeable. These results point to the importance of inducing dual identity under specific conditions for research on decreasing prejudice. Some practical implications of the findings for urbanization and immigration are discussed.

## Introduction

The world is undergoing a rapid urban transformation, and large numbers of the rural population are moving to urban areas. How to promote harmony between the host residents and migrants has received much research attention (Hewstone and Brown, 1986; Riek et al., 2006). Empirical research has shown that dual identity, simultaneous activation of both subgroup and superordinate group identities, is effective for increasing the majority group members’ positive attitude and behavior toward outgroups (e.g., Gaertner et al., 1996; Glasford and Dovidio, 2011; Scheepers et al., 2014). Dual identity works because it prevents group identity from being threatened by satisfying people’s need for distinctiveness while maintaining the beneficial effects of a common ingroup identity (Dovidio et al., 2007). People’s distinctiveness motivation is highly aroused when outgroup members have the opportunity to enter their ingroup, that is, when group boundary is permeable (Bettencourt et al., 2001; Cadinu and Reggiori, 2002; Verkuyten and Reijerse, 2008). However, people have a relatively low level of need for distinctiveness in the context where group boundary is impermeable (Sidanius and Pratto, 2001; Dovidio et al., 2007, 2009). Therefore, we supposed that group boundary permeability might exert a moderating role on the effect of dual identity on prejudice.

### Dual Identity and Prejudice

The common ingroup identity model (CIIM, Gaertner and Dovidio, 2000) proposes that intergroup bias can be reduced by inducing members of different groups to an inclusive, superordinate identity group because of the cognitive and motivational processes involving ingroup favoritism (Gaertner et al., 1993; Scheepers et al., 2014). However, a common identity may threaten the distinctiveness of subgroup identities and therefore exacerbate intergroup relationship (e.g., Hornsey and Hogg, 2000; Crisp et al., 2006a), and decrease the likelihood that majority group members recognize and respond to injustice (Saguy and Chernyak-Hai, 2012; Banfield and Dovidio, 2013).

Based on research on the mutual intergroup differentiation model (Hewstone and Brown, 1986; Brown and Hewstone, 2005), the dual identity model of re-categorization has been identified (Dovidio et al., 2007), which is considered an effective intervention for improving intergroup relationship. Dual identity not only recognizes subgroups’ differences but also creates an overarching category. For example, group members can conceive two distinctive groups (e.g., White and Black) within a superordinate (i.e., American) social identity. Dual identity can be promoted by simultaneously emphasizing subgroup identities and superordinate ingroup identity. Research has demonstrated that inducing a dual identity can promote the majority group members’ positive attitudes and action toward minority members (e.g., Gaertner et al., 1996; González and Brown, 2003, 2006; Guerra et al., 2010; Banfield and Dovidio, 2013; Scheepers et al., 2014). For example, research has demonstrated that creating dual identity reduces the bias of high-status majority group members (González and Brown, 2003, 2006; Guerra et al., 2010). Moreover, for White Americans, the endorsement of dual identity can facilitate greater recognition of bias and produce greater motivation to act for Black Americans (Banfield and Dovidio, 2013).

Dovidio et al. (2007) proposal suggested the important role of the need for distinctiveness in the positive effect of dual identity on out-group attitudes. Previous research has also found that ethnic minority members who need to maintain their identity distinctiveness have a more positive attitude toward the majority group members when a dual identity is induced compared to a control condition (Glasford and Dovidio, 2011). From this perspective, when group members have a need for distinctiveness, dual identity is effective in promoting intergroup attitude. When they do not have a need for distinctiveness, dual identity may not be effective.

### Group Permeability Moderates Impact of Dual Identity on Prejudice

We proposed that for majority group members, the permeability of group boundary could influence the effectiveness of dual identity to reduce their prejudice against minorities. Permeability refers to the extent to which individual group members can leave one group and join another (Tajfel and Turner, 1979, 1986). Permeable group boundaries imply possibilities for upward social mobility for disadvantaged groups, which present threats to the ingroup identity of the dominant group (Tajfel and Turner, 1979, 1986; Taylor and McKirnan, 1984; Echabe and Castro, 1996; Verkuyten and Reijerse, 2008). When the distinctiveness of group identity is threatened, people are motivated to secure their status advantage and pursue positive group distinctiveness (Deschamps and Brown, 1983; Mullin and Hogg, 1998; Hogg and Mullin, 1999; Bettencourt et al., 2001; Cadinu and Reggiori, 2002). In this condition, inducing dual identity can satisfy the majority people’s need for group distinctiveness and further reduce their prejudice against the minority group members. Thus, a high-status majority with dual identity will show less prejudice when the group boundary is permeable.

By contrast, the impermeability of group boundary may decrease the high-status majority’s need for distinctiveness. When the group boundary is impermeable, disadvantaged group members have fewer chances for joining the advantaged group, and the identity threat from minority to majority is low (Verkuyten and Reijerse, 2008). At this time, majority group members have a low need for distinctiveness (Bettencourt et al., 2001). Inducing dual identity that includes different subgroup identities makes intergroup differences salient, which leads to a negative outgroup attitude and eliminates the benefit of a superordinate identity. Thus, when the group boundary is impermeable, dual identity interventions will not be effective in reducing prejudice against the minority group. Taken together, we expected that group permeability would moderate the relationship between dual identity and prejudice.

### The Current Study

The present research was conducted with Chinese participants. In China, the *Hukou* system, which was formed in the late 1950s and refers to the household registration, is promulgated to manage residential groups and register population separately in rural and urban areas (Mallee, 2000; Guan and Liu, 2014). According to their permanent residence, individuals are registered as an urban category or a rural category. The *Hukou* system restricts the rural-to-urban migrants from settling in urban areas and deprives them of a wide range of basic welfare and government-provided services enjoyed by urban residents (Wing Chan and Buckingham, 2008; Cai, 2011). Hence, compared to the urban residents, the rural-to-urban migrants are seen as inferior, second-class citizens who experience prejudice and discrimination from urban residents (e.g., Kuang and Liu, 2012; Zhang et al., 2014).

We aimed to extend previous research by exploring the relationship of dual identity and prejudice within a new group and context. Previous research on dual identity and majority group members’ attitude against a minority outgroup was mainly conducted with groups divided by national identity or ethnicity (Gaertner et al., 1996; Crisp et al., 2006a; Guerra et al., 2010; Banfield and Dovidio, 2013; Scheepers et al., 2014), while urban residents and rural-to-urban migrants in China are two groups that have been divided by the social institution of *Hukou* for several decades. We hypothesized that the condition that emphasizes dual identity, affirming both *Hukou* categorization and superordinate group identity as Chinese, will produce less prejudice against rural-to-urban migrants than the control condition (H1).

The reform of the *Hukou* system aims to diminish the differences between the urban residents and rural-to-urban migrants and encourage rural-to-urban mobility (Mo, 2014), which provides a natural laboratory for exploring the moderating role of group boundary on the relationship between dual identity and prejudice. We manipulated group boundary permeability by describing whether the *Hukou* reform succeeded or not, and hypothesized that group permeability would moderate the relationship between dual identity and prejudice (H2).

To test the above hypotheses, Study 1 explored whether inducing dual group identity would decrease the majority group members’ prejudice by manipulating the salience of a dual identity, Study 2 further investigated the moderation role of group permeability on the relationship between dual identity and prejudice by experimentally manipulating the group boundary permeability and dual identity.

## Study 1

### Method

#### Ethics Statement

The study was reviewed and approved by the Committee of Protection of Subjects at Beijing Normal University. All participants provided written informed consent before the study and were debriefed at the end of the research according to the established committee guidelines. This procedure was also followed in Study 2.

#### Participants

Participants were 71 urban residents (47.1% women, one declined to report gender, *M* age = 26.44 years, *SD* = 5.56). None of the participants once were rural-to-urban migrants. In terms of educational backgrounds, among the participants, 45.1% of them held masters or doctoral degrees, 49.3% held bachelor’s degrees and the rest held high school diplomas. They were recruited for the study through an online survey and received 3 (approximately US$ 0.45) as monetary compensation for taking part in the short online survey. Participants were randomly assigned to one of two between-subjects manipulation conditions: dual identity (*n* = 36) or control (*n* = 35). A *post hoc* power analysis conducted using GPower (Faul et al., 2007) indicated that the achieved power of the effect of dual identity on prejudice approached to 80.6% in this study, suggesting the sample size was large enough.

#### Materials

##### Group identity manipulation

The group identity was manipulated by one of two articles, modeled after previous research on the CIIM (Glasford and Dovidio, 2011; Banfield and Dovidio, 2013). In dual identity condition, the news report was designed to make the common Chinese and subordinate (urban and rural *Hukou*) identity salient. The news report was read as follows: “We urban residents and rural-to-urban migrants are from two different groups, but we also belong to a common group, the Chinese. Recognizing that all of us are members of groups that have different traditions but also share a common Chinese identity can contribute to making a harmonious society. Social scientists propose that an approach that simultaneously emphasizes the differences between the urban residents and rural-to-urban migrants and our common identity as Chinese is an essential component to the well-being of both group members.” In the control condition, participants read a short news report on a subject that was about the exhibition plan of the Palace Museum. Both urban citizens and rural-to-urban migrants can visit Palace Museum. Stated differently, the entry into Palace Museum is not a privilege of urban citizens. Therefore, the control condition could not influence participants’ social identity.

##### Group identity manipulation check

The dual group identity was assessed by the item “Even though urban residents and rural-to-urban migrants are members of different groups, they both contribute to making China a better nation.” The participants were asked to choose a number from 1 (*completely disagree*) to 7 (*completely agree*) to indicate their level of agreement with the item. The item was adapted from the measure used by Glasford and Dovidio (2011). Higher score indicated higher level of dual identity participants possessed.

##### Prejudice

Prejudice against the rural-to-urban migrants was measured by the 9-item Social Distance Scale adapted from Bogardus (1925), which was used as an index of prejudice against rural-to-urban migrants (Kuang and Liu, 2012; Zhang et al., 2014, see in **Appendix**). On a 7-point Likert scale (1 = strongly disagree; 7 = strongly agree), the participants indicated the extent to which they endorsed the statement. The underlying assumption is that the more social distance one wants to hold to a certain group, the more prejudice the rater holds toward that group. A sample item was, “I would like to treat rural-to-urban migrants as friends” (reverse-scored; Cronbach’s α = 0.79).

#### Procedure

Participants were told that they would first read an article and then respond to some questions. They were randomly assigned to one of two conditions in which they read about news emphasizing dual identity (dual identity condition) or something neutral and unrelated to group identity (control condition). Then, participants proceeded to complete measures of dual group identity, prejudice toward rural-to-urban migrants, and demographics.

### Results and Discussion

#### Manipulation Check

We compared urban residents’ perception of dual identity across conditions. Participants primed with dual identity showed significantly higher scores on dual identity (*M* = 6.47, *SD* = 0.70) than those in the control identity condition (*M* = 5.91, *SD* = 1.04), *t*(69) = 2.66, *p* = 0.010, Cohen’s *d =* 0.63. This result established that our manipulation was successful.

#### Prejudice toward Rural-to-Urban Migrants

**FIGURE 1:**
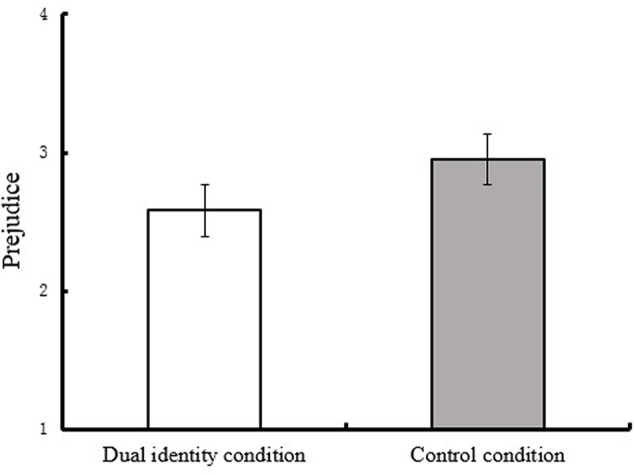
**Prejudice toward rural-to-urban migrants as a function of group identity (Study 1)**.

After the control of demographical variables (i.e., participants’ and their parents’ education background), the main effect for group identity on prejudice was significant, *F*(1,65) = 7.414, *p* < 0.01, $\eta_{p}^{2}$ = 0.102. Results (see **Figure 1**) revealed that participants in dual identity condition showed lower prejudice (*M* = 2.58, *SD* = 0.72) than those in the control condition (*M* = 2.95, *SD* = 0.72).

In order to examine whether there was gender difference on prejudice, a 2 (Group identity: dual identity condition, control condition) × 2 (Gender: male, female) ANOVA was conducted on prejudice. Results suggested that the main effect of gender on prejudice was marginally significant, *F*(1,66) = 2.85, *p* = 0.096, $\eta_{p}^{2}$ = 0.041, with female participants (*M* = 2.91, *SD* = 0.63) holding more prejudice than male participants (*M* = 2.62, *SD* = 0.81). Gender did not moderate the effect of group identity on prejudice, *F*(1,66) = 1.051, *p* = 0.309, $\eta_{p}^{2}$ = 0.016.

In sum, Study 1 provides evidence that inducing dual identity by emphasizing the subgroup identity (*Hukou* categorization) and superordinate identity (Chinese) is effective in reducing majority group members’ prejudice toward the minority group relative to a control condition. The pattern of findings is consistent with previous literature indicating that dual identity intervention is effective in reducing majority group members’ bias toward minority groups (González and Brown, 2003, 2006). Thus, the hypothesis set for Study 1 (H1) was confirmed.

## Study 2

Study 1 revealed that dual identity was effective in reducing urban residents’ prejudice against rural-to-urban migrants in China. In Study 2, we aimed to further explore whether the group boundary permeability moderates the effects of dual identity on prejudice against rural-to-urban migrants. The group boundary permeability was manipulated with the *Hukou* system reform in China.

### Method

#### Participants

Participants were 132 urban residents (54.5% women, *M* age = 25.21 years, *SD* = 5.27). None of the participants once were rural-to-urban migrants. In terms of the educational backgrounds, among the participants, 51.5% held masters or doctoral degrees, 46.2% bachelor’s degrees, and the rest held high school diplomas. They were recruited for the study through an online survey and received 3 (approximately US$ 0.45) as monetary compensation for taking part in the short online survey.

#### Materials

##### Group boundary permeability manipulation

Group boundary permeability was manipulated by one of two different online news, which was about rural-to-urban migrants being able to attain a non-agricultural household or not, modeled after Zhang et al. (2014). In the permeable condition, participants read a piece of news telling them that a new *Hukou* policy enabled more and more rural-to-urban migrants to obtain non-agricultural *Hukou* status in Guangzhou. However, in the impermeable condition, the news revealed that due to the failure of the new policy, rural-to-urban migrants still could not obtain non-agricultural *Hukou* status in Guangzhou. It was also mentioned in the materials that Guangzhou was a trial city of household system reform, the reform condition that reflects the overall process of rural-to-urban migrants’ urbanization in China.

##### Group boundary permeability manipulation check

The group permeability was measured using the following two items (Zhang et al., 2014): “What is the probability for rural-to-urban migrants to become urban residents?” and “How easily do rural-to-urban migrants become urban residents?” Participants were asked to choose a number from 1 (*completely disagree*) to 7 (*completely agree*) to indicate their level of agreement with each item. The two items were highly positively correlated, *r*(132) = 0.500, *p* < 0.01. The average scores of the two items were regarded as an index of group permeability. Higher scores indicated greater perceived group permeability.

##### Dual identity manipulation

Following the manipulation of group permeability, the participants were randomly assigned to dual identity or control condition. As in Study 1, participants either read a news article about a museum exhibition (control condition) or read a news article that emphasized the common Chinese and salient subordinate (urban and rural *Hukou*) identity (dual identity condition).

##### Dual identity manipulation check

As in Study 1, dual identity was measured by the item, “Even though urban residents and rural-to-urban migrants are members of different groups, they both contribute to making China a better nation.”

##### Prejudice

The social distance scale (same as in Study 1) was used to measure participants’ prejudice against rural-to-urban migrants. The items were rated on a 7- point scale ranging from 1 (completely disagree) to 7 (completely agree). Higher scores represented stronger prejudice against rural-to-urban migrants. The average score of the nine items was calculated as a prejudice indicator (α = 0.89).

#### Procedure

Participants were randomly assigned to the various conditions in a 2 (Group Boundary Permeability: permeable and impermeable) × 2 (Group Identity: dual group identity and control condition) between-subjects design. Similar to Study 1, they were informed that they would read and respond to several articles. After reading the news, participants completed a set of questionnaires, including some items concerning the details in the news, manipulation check, social distance scale, and demographics.

### Results and Discussion

#### Manipulation Check

First, the permeability index was submitted to a 2 (Group Boundary Permeability: permeable and impermeable) × 2 (Group Identity: dual-identity and control condition) ANOVA. Results revealed that the main effect of group permeability manipulation on permeability was significant, *F*(1,128) = 7.40, *p* < 0.01, *$\eta_{p}^{2}$* = 0.055. As expected, participants in the permeable condition perceived higher permeability (*M* = 2.26, *SD* = 0.84) than participants in the impermeable condition (*M* = 1.85, *SD* = 0.86). The main effect of identity manipulation [*F*(1,128) = 0.089, *p* = 0.766, $\eta_{p}^{2}$ = 0.001] and the two-way interaction [*F*(1,128) = 0.22, *p* = 0.638, $\eta_{p}^{2}$ = 0.002] were not significant.

Moreover, dual identity index was submitted to the same ANOVA. The main effect of group identity manipulation on dual identity was significant, *F*(1,128) = 3.88, *p* = 0.051, $\eta_{p}^{2}$ = 0.029. Participants under dual identity condition showed significantly higher scores on dual identity (*M* = 6.34, *SD* = 0.89) than did those in the control condition (*M* = 5.97, *SD* = 1.09). Neither the main effect of group permeability [*F*(1,128) = 0.49, *p* = 0.484, *$\eta_{p}^{2}$* = 0.004] nor the two-way interaction [*F*(1,128) = 0.41, *p* = 0.52, $\eta_{p}^{2}$ = 0.003] was significant. The results suggested that our manipulations of group permeability and group identity were successful.

#### Prejudice toward Rural-to-Urban Migrants

The 2 (Group Boundary Permeability: permeable and impermeable) × 2 (Group Identity: dual-identity and control condition) analysis of variance (ANOVA), which was conducted on participants’ prejudice toward rural-to-urban migrants, revealed a main effect for group identity that was marginally significant, *F*(1,128) = 3.37, *p* = 0.069, $\eta_{p}^{2}$ = 0.026. Overall, urban residents showed less prejudice against rural-to-urban migrants in dual identity condition (*M* = 2.49, *SD* = 0.85) than those in control condition (*M* = 2.82, *SD* = 0.90). However, this analysis showed no significant main effect for the manipulation of group boundary permeability, *F*(1,128) = 1.64, *p* = 0.202, $\eta_{p}^{2}$ = 0.013.

**FIGURE 2:**
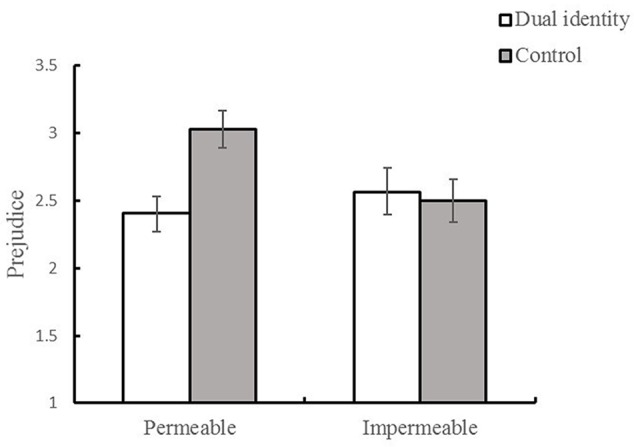
**Prejudice toward rural-to-urban migrants as a function of group identity and group permeability (Study 2)**.

Consistent with our main prediction, this effect was moderated by group boundary permeability: the Group Boundary Permeability × Group Identity interaction was significant, *F*(1,128) = 5.01, *p* = 0.027, $\eta_{p}^{2}$ = 0.038 (see **Figure 2**). Simple effects analyses revealed that when group boundary was permeable, urban residents showed significantly lower prejudice in dual identity condition (*M =* 2.41, *SD =* 0.70) than those in the control condition (*M* = 3.03, *SD* = 0.88), *F*(1,129) = 9.77, *p* < 0.01, **$\eta_{p}^{2}$** = 0.13. However, when group boundary was impermeable, participants in dual identity condition showed equivalent prejudice as those in the control condition, *M =* 2.56 (*SD =* 1.00) versus *M =* 2.50 (*SD =* 0.84), respectively; *F*(1,129) = 0.04, *p* = 0.849, $\eta_{p}^{2}$ = 0.001. Alternatively stated, in the control condition, participants showed more prejudice in the permeable condition than did those in the impermeable condition, *F*(1,129) = 7.50, *p* < 0.01, $\eta_{p}^{2}$ = 0.086. In dual identity condition, group boundary permeability had no such effect, *F*(1,129) = 0.48, *p* = 0.49, $\eta_{p}^{2}$ = 0.007.

In order to examine whether there was a gender difference on prejudice, a 2 (Group Boundary Permeability: permeable and impermeable) × 2 (Group Identity: dual-identity and control condition) × 2 (Gender: male and female) ANOVA was conducted on prejudice. Results suggested that the main effect of gender on prejudice was significant, female participants (*M =* 2.81, *SD =* 0.90) showed more prejudice against rural-to-urban migrants than male participants (*M =* 2.47, *SD =* 0.85), *F*(1,124) = 4.718, *p* = 0.032, $\eta_{p}^{2}$ = 0.037. The absence of the three-way interaction [*F*(1,124) = 0.311, *p* = 0.578, $\eta_{p}^{2}$ = 0.002] suggested that gender did not moderate the interaction effect between group identity and permeability condition on prejudice. In order to examine whether gender differences in prejudice could be partially accounted for by educational differences, we used a bootstrapping procedure (Hayes, 2013) to estimate the mediational role of education in the relationship between gender and prejudice. Results suggested that there was no gender differences in educational level (*B* = 0.013, *SE* = 0.056, *p* = 0.224) and prejudice could not be explained by participants’ educational levels (*B* = -0.175, *SE* = 0.120, *p* = 0.148). In conclusion, participants’ educational differences could not explain the effect of gender on prejudice (*B* = -0.002, *SE* = 0.014; CI = LL: -0.045; UL: 0.016). Alternatively, we suppose the gender difference on prejudice may result from the negative stereotype of rural-to-urban migrants. Rural-to-urban migrants are stigmatized by urban residents as potential perils of danger and crime (Guan and Liu, 2014). Because of the stigma, female participants may perceive more security threat from rural-to-urban migrants than male participants. Hence, female participants showed more prejudice against rural-to-urban migrants.

Consistent with our prediction, when the group boundary was permeable, participants with dual identity showed significantly lower prejudice than did those in the control condition. By contrast, when the group boundary was impermeable, dual identity was no longer effective in reducing prejudice. This result provided direct experimental support for previous work asserting that the cultural and political context may influence the meaning of group identity (Dovidio et al., 2009).

## General Discussion

Across two experiments, we extended previous research on dual identity and prejudice by exploring this relationship in a new group and context, and by identifying a boundary condition in this relationship, that is, group permeability. The results supported our two hypotheses: First, the condition that emphasizes dual identity, affirming both *Hukou* categorization and common group identity as Chinese, will produce less prejudice against rural-to-urban migrants than that in the control condition (H1). Second, group permeability moderates the effect of dual identity on prejudice against rural-to-urban migrants (H2).

We found that dual identity was effective in reducing urban residents’ prejudice against rural-to-urban residents. This result thus verifies the validity of dual identity intervention on improving attitude toward relevant outgroups (González and Brown, 2003, 2006). Moreover, it extends existing research on dual identity and prejudice to a new group and context consisting of urban residents in China. Most of the previous research has been conducted with groups divided by national identity (e.g., Crisp et al., 2006b) or with groups divided by ethnicity (e.g., Guerra et al., 2010; Scheepers et al., 2014). In our case, urban residents and rural-to-urban migrants are two groups that have been divided by the *Hukou* registration policy for more than 60 years.

Building on previous research that dual identity can satisfy people’s need for distinctiveness to reduce prejudice, and that group permeability can influence the majority people’s need for distinctiveness (Bettencourt et al., 2001; Dovidio et al., 2007, 2009; Verkuyten and Reijerse, 2008), the present research demonstrates that when the group boundary is permeable, urban residents with dual identity produce less prejudice against rural-to-urban migrants. However, when the group boundary is impermeable, dual identity is not effective in reducing prejudice.

Our results make an important contribution to the literature on dual identity and prejudice. We highlight the importance of the social context factor, group permeability, for moderating the association of dual identity and the majority members’ prejudice against migrants. Previous research has found that the stability and legitimacy of group status affected the majority group members’ identity preference (Saguy and Dovidio, 2013), which may further influence the effectiveness of identity intervention to facilitate intergroup relationship (Dovidio et al., 2015). However, these studies did not sufficiently explore the influence of sociostructural factors on the effectiveness of dual identity to reduce prejudice. In our research, compared to previous studies, we explored the effects of group permeability on this relationship and added a control group. Our findings also complement the claim that different historical, cultural, and political contexts could influence the functions of different kinds of group identity (Dovidio et al., 2009). Dual identity is more effective to reduce intergroup prejudice when the intergroup mobility brings up stronger distinctiveness motivation for the advantaged group members. In contrast, when intergroup mobility is restricted and the priority of the advantaged group is fully recognized, common identity may be more effective to reduce prejudice because it highlights similarities rather than differences between groups.

From a practical perspective, our result indicates that when dual identity intervention is employed to reduce prejudice, the social context factor such as group permeability should be considered. The developing world is witness to a rapid urbanization, immigration and social integration. The upward mobility of members of disadvantaged and minority groups or migrants may elicit more threat of distinctiveness for advantaged and dominant group members. Therefore, if the permeable group boundary is inevitable, measures or policies stressing both intergroup differences and similarities should be made. Surely, the permeability of intergroup boundary may vary across different groups. To facilitate intergroup harmony, the employment of dual identity, common identity or other strategies should adjust to specific condition and background.

There are several limitations of the present work that offer opportunities for future research. First, we examined the urban residents’ prejudice against rural-to-urban migrants, not their actual behavior. Collective action by high-status group members that aims to help low-status group members achieve equality may be more effective than that by low-status group, because high-status group members possess more resources and face less resistance from other majority group members. Future research should investigate urban residents’ collective action for rural-to-urban migrants. Second, although we confirmed that the manipulation of group permeability influenced the group’s need for distinctiveness, we did not assess this variable directly. Thus, although the present research found that group permeability moderated the relationship between dual identity and the majority members’ prejudice against migrants, the precise mechanisms remain unclear. Future research should examine the mediators for this relationship. Third, we found that in the group-impermeable condition, inducing dual identity did not decrease the majority’s prejudice against the rural-to-urban migrants. However, we did not explore which kind of group identity can decrease the prejudice against rural-to-urban migrants in the condition where group boundary is impermeable. Because the impermeable group boundary made the majority members have a low need for distinctiveness (Saguy and Dovidio, 2013), we assume that the common identity will be effective for reducing prejudice against migrants in this condition, which can be examined in future research.

## Conclusion

With the present research, we further explored the effectiveness of dual identity to reduce prejudice and the moderating role of group permeability on this relationship. We can draw two main conclusions: First, inducing dual identity can significantly reduce urban residents’ prejudice against rural-to-urban migrants. Second, group permeability moderates the relationship between dual identity and prejudice. Only when the group boundary is permeable, which refers to the *Hukou* system reform, is dual identity effective in reducing urban residents’ prejudice against rural-to-urban migrants.

## Author Contributions

The first author, YS, contributed to all aspects of work for this article. LL contributed to conception, design and revising the article critically. JD contributed to data collection, design and interpretation. WZ contributed to interpretation and revising the article carefully.

## Conflict of Interest Statement

The authors declare that the research was conducted in the absence of any commercial or financial relationships that could be construed as a potential conflict of interest.

## APPENDIX

Adapted Social Distance Scale

(1) Rural-to-urban migrants appear to be likeable persons.
(2) I would like rural-to-urban migrants to be close friends.
(3) I would not mind it at all for rural-to-urban migrants to move into my neighborhood.
(4) I would like rural-to-urban migrants to come and work at the same place I do.
(5) I would feel uncomfortable when rural-to-urban migrants are around.
(6) Rural-to-urban migrants are the kind of person that I tend to avoid.
(7) I would like to talk with rural-to-urban migrants.
(8) I would like to rent my house to rural-to-urban migrants.
(9) I would like rural-to-urban migrants to join in the management of community.
